# A Case of Pheochromocytoma Presenting With Acute Coronary Syndrome

**DOI:** 10.7759/cureus.61389

**Published:** 2024-05-30

**Authors:** Jagannath S Dhadwad, Ramiz S Kadiwala, Sheetal N Kishore, Anish Chitnis, Dhairya Sanghani

**Affiliations:** 1 General Medicine, Dr. Dnyandeo Yashwantrao Patil Medical College, Hospital and Research Centre, Dr. Dnyandeo Yashwantrao Patil Vidyapeeth (Deemed to be University) Pune, Pune, IND

**Keywords:** resistant hypertension, benign adrenal neoplasm, incidentaloma, acute coronary syndrome, pheochromocytoma

## Abstract

Pheochromocytoma is a rare endocrine tumor originating from chromaffin cells of the adrenal medulla, which leads to the overproduction of catecholamines. Most symptoms, ranging from simple headaches to life-threatening cardiac arrests, are due to excess catecholamines. Usually, patients present with persistent or paroxysmal hypertension, headaches, sweating, and palpitations. Here, we describe a case that initially presented as an acute coronary syndrome and was treated accordingly. However, she had a history of nocturnal awakenings and panic attacks, which she had ignored for a month. On further evaluation, it turned out to be pheochromocytoma. This case report will surely help physicians better diagnose and treat such cases.

## Introduction

Pheochromocytomas are rare catecholamine-secreting neoplasms arising from chromaffin cells of the adrenal medulla. Clinical presentations are different according to location and extent of involvement. Classically, patients present with episodic headaches, sweating, and tachycardia [1]. The most common presentation is sustained or paroxysmal hypertension, but approximately 15%of patients may be normotensive [2]. Other symptoms include generalized weakness, pallor, tremors, dyspnea, and panic-attack-like symptoms. Cardiac manifestations include myocardial ischemia, arrhythmias, acute myocardial infarction, and decompensated heart failure [3]. With the extended availability of ultrasonography and computed tomography (CT), more and more cases are detected in the pre-symptomatic phase. The diagnosis of pheochromocytoma requires laboratory confirmation of excessive catecholamine production and radiological documentation. Most symptoms resolve after surgical resection of the neoplasm.

## Case presentation

**Figure 1:**
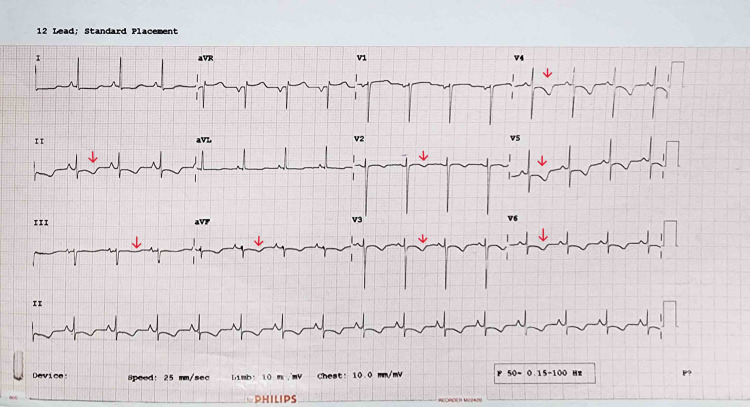
ECG suggestive of T-wave inversions in lead 2, 3, aVF, and pre-cordial leads V2-V6 (marked by red arrow). ECG: electrocardiogram

A 51-year-old female came to the casualty with an acute onset of sharp, constricting chest pain since midnight, which was progressive, radiating to the left shoulder, and accompanied by palpitations and dyspnea worsening on exertion. The electrocardiogram (ECG) showed T-wave inversions in leads 2, 3, aVF, and chest leads V2 to V6 (Figure 1), and cardiac biomarkers were elevated (Table 1). The patient was treated conservatively for acute coronary syndrome (ACS) and shifted to the ward for observation. She had a history of frequent nocturnal awakenings with palpitations and panic attack-like episodes lasting 10-15 min for 1 month. There were no known comorbidities. On presentation, her blood pressure was 180/100 mmHg in the supine position, pulse rate - 110/min, respiratory rate - 18/min, and her oxygen saturation was 96% on room air. The rest of the general and systemic examinations were normal. Initial investigations are in Tables 1-2.

**Table 1:** Blood investigations on day 1 and day 3 with reference ranges TLC: total leucocyte count; MCV: mean corpuscular volume; ANC: absolute neutrophil count; INR: international normalized ratio; aPTT: activated plasma thromboplastin time; PCV: packed cell volume; HDL: high-density lipoprotein; VLDL: very low-density lipoprotein; LDL: low-density lipoprotein; CK-MB: creatine kinase-myoglobin binding; fL: femto liter; mg/dL: milligram per deciliter; pg/dL: picogram per deciliter; mmol/L: millimoles per liter

Investigations	Day 1	Day 3	Reference Range
Hemoglobin	11.30	11.40	13.2-16.6 g/dL
TLC	6700	6400	4000-10000/µL
ANC	4900	4100	2000-7000/µL
Platelets	364000	366000	150000-410000/µL
MCV	88.70	88.90	78.2-97.9 fL
Total bilirubin	1.10	-	0.22-1.20 mg/dL
Direct bilirubin	0.35	-	< 0.5 mg/dL
Aspartate transaminase	34	-	8-48 units/L
Urea	56	44	17-49 mg/dL
Serum creatinine	2.02	1.30	0.6-1.35 mg/dL
INR	1.00	-	0.85-1.15
aPTT	26.0	-	21.75-28.70 sec
Serum sodium	140	141	136-145 mmol/L
Serum potassium	4.50	4.20	3.50-5.10 mmol/L
Serum cholesterol total	-	154	<200 mg/dL
Serum triglycerides	-	86	<150 mg/dL
HDL cholesterol	-	48	>40 mg/dL
VLDL cholesterol	-	26	<30 mg/dL
LDL cholesterol	-	80	<100 mg/dL
Troponin I	770	205	<10 pg/dL
CK-MB	114	44	<35 units/L

**Table 2:** Urine microscopy test RBC: red blood cells; hpf: high-power field

Parameter	Day 1	Reference range
Protein	1+	Absent
Glucose	2+	Absent
RBCs	Absent	0-2 cells/hpf
Pus cells	1-2	0-5/hpf
Urinary casts or crystals	Absent	Absent

**Figure 2:**
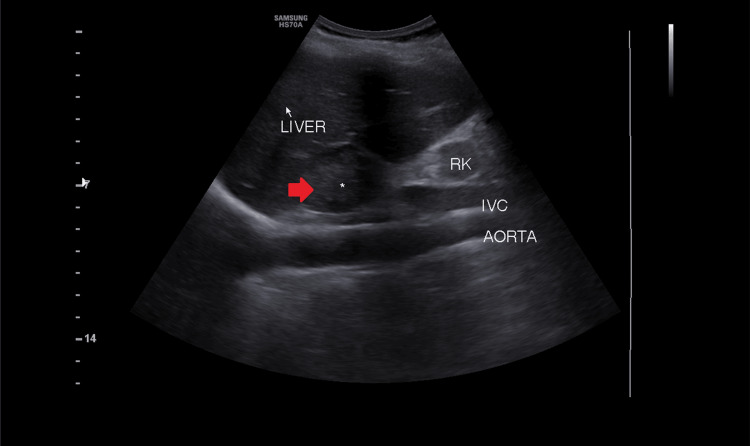
Ultrasonography of the abdomen showing a well-defined heterogeneously hypodense lesion (red arrow) measuring 36 mm x 32 mm in the right suprarenal region indenting right lobe of liver. Red arrow pointing to hypodense mass over the right kidney. RK: right kidney; IVC: inferior vena cava; mm: millimeter

**Figure 3:**
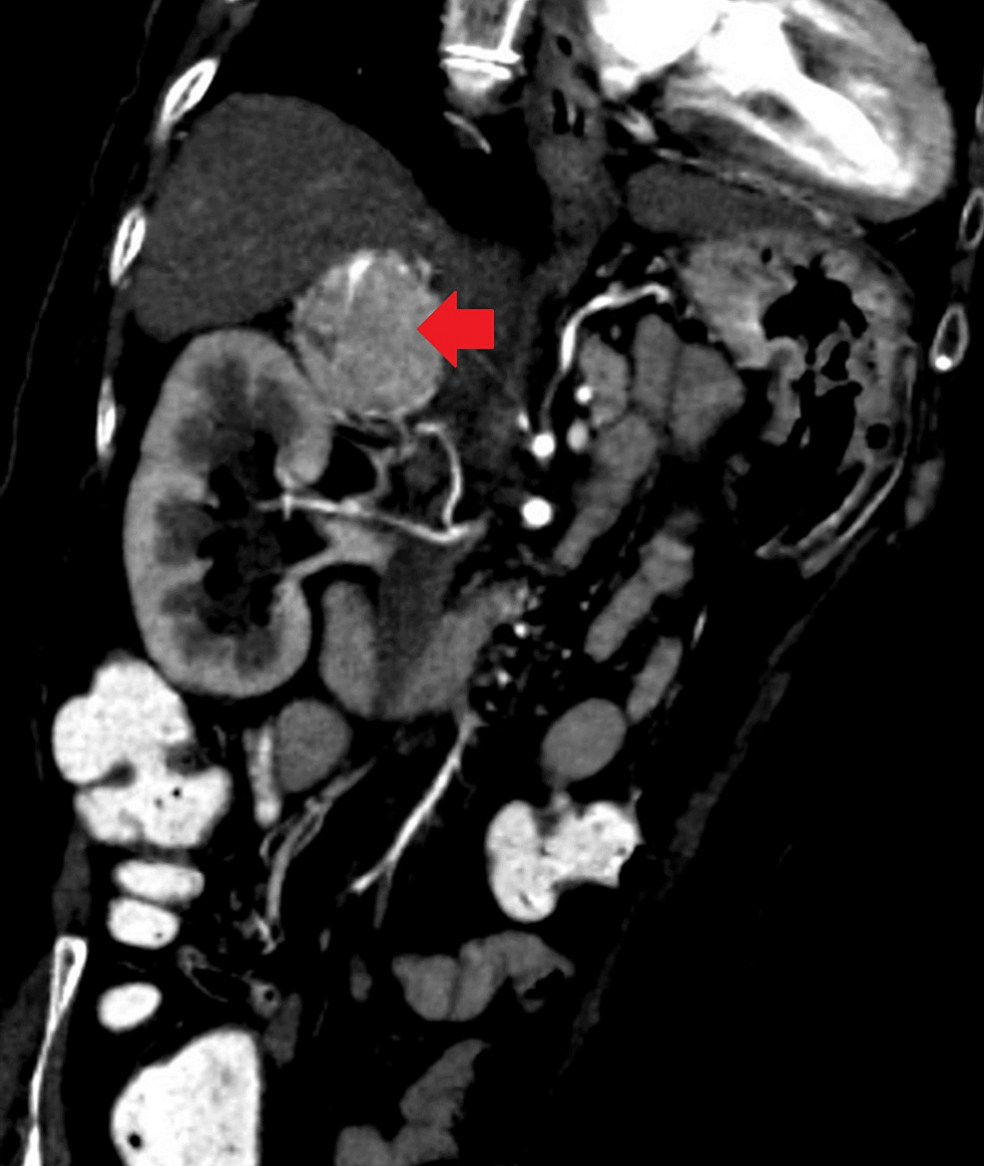
CECT of the abdomen showing well-defined iso-hypodense suprarenal lesion of size 45 mm x 40 mm x 35 mm arising from right adrenal gland marked by a red arrow. A well-defined iso-hypodense lesion arising from the right adrenal, located superior and anterior to the upper pole of the right kidney. The lesion is encased by the right suprarenal artery and located posterior to the inferior vena cava, compressing and displacing it anteriorly. CECT: contrast-enhanced computed tomography; mm: millimeter

Screening ultrasonography of the abdomen and pelvis revealed a heterogeneously hypodense lesion in the right suprarenal region (Figure 2). The diagnosis of pheochromocytoma looked likely. contrast-enhanced CT (CECT) of the abdomen confirmed a well-defined iso-hypodense suprarenal lesion measuring 45 mm x 40 mm x 35 mm arising from the right adrenal gland, explained in Figure 3.

Further laboratory investigations showed elevated levels of 24-hour urinary vanillylmandelic acid (VMA) and 24-hour urinary metanephrines (Table 3).

**Table 3:** Specific blood investigations for pheochromocytoma evaluation VMA: vanillylmandelic acid; mg/mL: milligram per milliliter; μg/L: microgram per liter

Investigations	Value	Reference Range
24-hour urinary VMA	28.56	<8.0 mg/mL
24-hour urinary metanephrines	491	<350 μg/L

Positron emission tomography (PET)-CT whole body was done to look for metastasis, which showed no adjacent spread. The patient was started on tab. prazosin 2.5 mg; the dose was increased to 5 mg twice daily. Sodium chloride capsules were added, and she was given a high-salt diet. Four days later, she was started on tab. metoprolol 50 mg. The patient underwent right adrenalectomy a week later. She was discharged on tab. Metoprolol 12.5 mg once daily and tab. Ramipril 2.5 mg once daily. Six months later, she comes for routine follow-ups; her nocturnal awakenings and panic attacks are treated. She has not had a single episode of nocturnal awakening or panic attack since the resection of the adrenal tumor.

## Discussion

Catecholamine-secreting neoplasms are rare, with an incidence of around one to four per million population per year [4]. They are mostly benign neoplasms, either arising from chromaffin cells of the adrenal medulla known as pheochromocytoma, or sympathetic ganglions known as paragangliomas. Pheochromocytomas are more commonly seen in the fourth and fifth decades but may occur at any age without any gender predilection [5]. As seen in this case, there are multiple reported cases with similar presentations with raised troponin levels [6-8]. One retrospective study among diagnosed cases of pheochromocytoma suggested that pheochromocytoma should be added as a differential diagnosis of ACS because the sudden release of catecholamines can induce chest pain and produce ECG changes. The study also noted that right-sided pheochromocytomas usually have more striking features [3]. Pheochromocytomas are mostly sporadic, but some cases have genetic bases or familial syndromic associations like von Hippel-Lindau (VHL) syndrome, neurofibromatosis type-1 (NF-1), and multiple endocrine neoplasia type-2 (MEN-2) [9]. Therefore, genetic testing is advisable for patients with a family history or early presentation of disease. The diagnosis of pheochromocytoma is suspected in patients with episodic headaches, palpitations, sweating, or paroxysmal hypertension. But around 58% of asymptomatic cases are detected incidentally during routine scans [10]. This makes the early diagnosis more tricky and requires a high degree of suspicion. Symptoms of pheochromocytoma occur due to catecholamines like norepinephrine, dopamine, and other immediate precursors of norepinephrine. So for diagnosis, we usually send 24-hour urinary and plasma fractionated metanephrines and catecholamines. Radiologically, a CT or MRI of the abdomen and pelvis is done as a first-line investigation or as a supportive investigation. Additionally, if required, a Ga-68 DOTATATE PET (Gallium-68 DOTATATE-positron emission tomography) or fludeoxyglucose-positron emission tomography (FDG-PET) scan is done in patients with a high degree of clinical suspicion in whom previous investigations were inconclusive. The only definitive treatment is surgical resection (adrenalectomy), but there is a high risk of intraoperative hypertensive crisis and arrhythmias, so patients should undergo pre-operative medical preparation [11]. Medical therapy is aimed at controlling blood pressure and correcting intravascular volume contraction. Alpha-adrenergic blockage (with phenoxybenzamine, prazosin, or doxazosin) for at least a week is given for normalization of blood pressure and volume expansion. Physicians should note that if beta-adrenergic blockers are to be added, they should only be added after a complete alpha-adrenergic blockade, as they may lead to a further increase in blood pressure if given before [12]. A high-salt diet (more than 5 g daily) is advised to aid volume expansion and counter orthostasis due to alpha-adrenergic blockade. Calcium channel blockers and metyrosine (a catecholamine synthesis inhibitor) are also added if blood pressure is not well controlled [13].

## Conclusions

Any patient presenting with episodic symptoms of headache, palpitations, sweating, and fluctuating blood pressure levels should be evaluated for pheochromocytoma. Simple ultrasonography or non-contrast CT (NCCT) of the abdomen is a good screening tool. In our case, the patient presented with ACS, which had many overlapping symptoms with pheochromocytoma. The patient had ignored the nocturnal awakenings and panic attack-like symptoms; only through history-taking and examination could we get a clue about pheochromocytoma. A high degree of suspicion, detailed history-taking, and thorough examination of the patients presenting with such symptoms is a must for treating physicians, especially when symptoms are episodic.
